# Insulin-like Growth Factor 1 (IGF1), IGF Binding Protein-3 (IGFBP3) and Growth Response to Daily Zinc Supplementation: A Randomized Trial in Rural Laotian Children

**DOI:** 10.3390/nu15112590

**Published:** 2023-05-31

**Authors:** Maxwell A. Barffour, Robin M. Bernstein, Guy-Marino Hinnouho, K. Ryan Wessells, Charles D. Arnold, Sengchanh Kounnavong, Sonja Y. Hess

**Affiliations:** 1 Department of Nutrition and Institute for Global Nutrition, University of California, Davis, CA 95616, USA; 2 Department of Medicine, University of Missouri School of Medicine, Columbia, MO 65212, USA; 3 Public Health Program, McQueary College of Health and Human Services, Missouri State University, Springfield, MO 65897, USA; 4 Department of Anthropology, University of Colorado, Boulder, CO 80309, USA; 5 Health and Society Program, Institute for Behavioral Science, University of Colorado, Boulder, CO 80309, USA; 6 Helen Keller International, Washington, DC 20006, USA; 7 Lao Tropical and Public Health Institute, Ban Kaognot, Sisattanack District, Vientiane 01030, Laos

**Keywords:** IGF1, IGFBP3, zinc supplementation, multiple micronutrient powder, physical growth

## Abstract

Objectives: To assess (a) the impact of daily preventive zinc tablets (7 mg; PZ), zinc-containing multiple micronutrient powder (10 mg zinc, and 13 other micronutrients; MNP) or placebo, delivered for 9 months, on Insulin-like Growth Factor 1 (IGF1) and IGF Binding Protein 3 (IGFBP3) among Laotian children 6–23 months, and (b) whether the effects of PZ and MNP on length-for-age z-scores (LAZ) and weight-for-age z-scores (WAZ) are modified by baseline IGF1 and IGFBP3. Design: A double-blind, placebo-controlled trial (N = 419). Methods: Plasma IGF1 and IGFBP3 concentrations at baseline and 36 weeks were analyzed by automated chemiluminescent assay. Anthropometry was assessed at baseline, at 18 and 36 weeks. Intervention effects were estimated using ANCOVA. Results: At 36 weeks, geometric mean IGF1 (~39.0–39.2 ng/mL; *p* = 0.99) and IGFBP3 (2038–2076 ng/mL; *p* = 0.83) did not differ by group. At 18 weeks (but not at 36 weeks), LAZ in the PZ group (−1.45) was higher than the MNP (−1.70) and control (−1.55) groups (*p* = 0.01) among children in the highest baseline IGF1 tertile (*p* for interaction = 0.006). At 36 weeks (but not at 18 weeks), WAZ in the PZ group (−1.55) was significantly higher than the MNP (−1.75) and control (−1.65) groups (*p* = 0.03), among children in the lowest baseline IGFBP3 tertile (*p* for interactions = 0.06). Conclusions: Although IGF1 and IGFBP3 did not respond to PZ and MNP, baseline IGF1 and IGFBP3 significantly modified the impact of PZ on linear and ponderal growth, suggesting that IGF1 bioavailability may drive catch-up growth in zinc-supplemented children

## 1. Introduction

Growth faltering, including both linear and ponderal growth restriction, is a major public health problem among children in settings with poor nutrition [1]. A growing body of epidemiological evidence suggests that zinc deficiency may be implicated in linear growth faltering. Zinc modulates cell replication and differentiation, and hence may affect physical growth status [2]. Several systematic reviews have concluded that preventive zinc supplementation (PZ), when delivered as a single nutrient supplement, improves linear growth among children in low- and middle-income countries [3,4,5,6]. However, the systematic reviews found a high degree of heterogeneity, with evidence of no impact in some settings. There is therefore a need for additional research to shed light on the particular contexts where zinc supplementation may be beneficial.

Furthermore, because of the coexistence of multiple micronutrient deficiencies among children presenting with growth stunting, there is a growing interest in interventions delivering multiple micronutrients concurrently, such as multiple micronutrient powder (MNP) recommended to improve iron status and anemia among infants and young children 6–23 mo of age by the World Health Organization (WHO) and UNICEF [7]. In contrast to intervention trials providing single-nutrient zinc supplements, trials of standard MNP formulations delivering 4.1–5 mg/d of zinc have failed to produce a measurable impact on growth [8,9], possibly because of interaction between zinc and iron, or the fact that MNP is typically consumed with phytate-rich foods, which limit zinc absorption [10]. Growth assessments are typically based on absolute or standardized indicators of height and weight. However, there are growing concerns that these conventional anthropometry indicators may not be sensitive enough to capture moderate to small growth changes occurring over a short period of time [11]. Biochemical indicators of growth may be an alternative, complementary approach for defining growth status and growth potential [11]. Hence, the goal of this study was to assess potential changes in growth biomarkers among children who did and did not receive supplemental zinc.

Growth hormone (GH), produced by the anterior pituitary gland, is the primary regulator of anabolism and growth [12,13]. It is postulated that growth hormone promotes linear growth by stimulating dividing prechondrocytes in the epiphyseal plate [14,15]. Because the secretion of GH is pulsatile, with large diurnal variations in circulatory levels [16], the measurement of GH and the assessment of its association with growth in epidemiological studies is challenging. Therefore, there is interest in the use of other markers of the growth hormone-insulin-like growth factor-1 (GH-IGF1) axis in characterizing growth status, growth potential, and the response in growth to preventive and therapeutic interventions. Of the IGF family of proteins, IGF1 is of particular interest because of its direct involvement in mediating the effects of GH, by promoting the clonal expansion of prechondrocytes primed by GH [17]. In addition, IGF1 levels, unlike GH, are stable in healthy individuals [18], and emerging evidence suggests that IGF1 directly stimulates growth, independent of GH stimulation [19,20].

The majority of IGF1 in circulation is found in ternary complexes of IGF1, IGF binding protein 3 (IGFBP-3) and an acid-labile subunit, which together influences the bioavailability of IGF1, and therefore, its effects on growth [21]. Unfortunately, epidemiological studies relating IGF1 and its binding protein to growth are few, and there is inconclusive evidence on whether IGF1 and IGBP3 moderate the growth-promoting effects of zinc [22,23]. In malnourished Bangladeshi children 6–36 months old and undergoing nutritional rehabilitation, supplementation for up to 30 days with different zinc regimens had no impact on IGF1 nor its binding proteins IGFBP-2 and IGFBP-3 [22]. In contrast, in malnourished Vietnamese children zinc supplementation was associated with higher IGF1 levels, during 5 months of daily supplementation [23].

To expand understanding of the role of the GH-IGF1 axis in mediating the growth effects of zinc, the present study was implemented in the context of a community-based randomized controlled trial designed to assess, among other things, the effects of different zinc supplementation strategies on physical growth among young children in rural Lao People’s Democratic Republic (Lao PDR). The main study outcomes were presented elsewhere [24,25]. Briefly, despite improving plasma Zn concentration, daily supplementation of young children for 9 months with either single-nutrient zinc supplement or zinc-containing MNP had no impact on linear growth and the prevalence of diarrhea and acute respiratory tract infections [24,25]. In this paper, we aimed to assess the effects of daily supplementation for 36 weeks with a single-nutrient zinc supplement or zinc-containing MNP on IGF1, IGFBP3, and IGF1 bioavailability as defined by the molar IGF1:IGFPB3 ratio. An additional study aim was to assess whether baseline IGF1, IGFBP3, and IGF1 bioavailability modified the intervention effect on length-for-age (LAZ) and weight-for-age z-scores (WAZ).

## 2. Methods

### 2.1. Ethical Approval

This trial was approved by the National Ethics Committee for Health Research (NECHR), Ministry of Health, Lao PDR, and the Institutional Review Board of the University of California, Davis, USA. The trial, known as as the Lao Zinc Study, was registered as NCT02428647 (https://clinicaltrials.gov (accessed on 30 May 2023)).

### 2.2. Study Design and Participants

In the parent study, a double-blind, placebo-controlled trial, children 6–23 months at enrollment were individually randomized to one of four intervention groups and followed for ~36 weeks to assess responses in physical growth [26]. The study was implemented from September 2015 through April 2017 in rural communities in Khammouane Province, central Lao PDR. The province was selected because of a high prevalence of stunting among children <5 years [27], the likely high risk of zinc deficiency, and no ongoing programs designed to reduce micronutrient deficiencies or treat diarrhea with therapeutic zinc supplements [26].

### 2.3. Sample Size Considerations

We estimated that a sample size of 140 per group would enable the detection of an effect size of 0.5 (90% power and 5% type 1 error rate) for the comparison of differences in IGF1 or IGFBP3 concentration between any 2 groups. Given the limited literature on the effect of micronutrient supplementation on these aforementioned biomarkers, this sample size was informed by the estimated effect size of the intervention on other biomarkers, such as plasma zinc and ferritin.

### 2.4. Inclusion and Exclusion Criteria

Children were considered eligible to participate in the study if they were 6–23 months of age at enrollment, their families intended to stay in the study area for the duration of the study, were willing to accept weekly home visits, and at least one of the caregivers (mother, father, legal guardian) provided written informed consent. Children were excluded from participation if they demonstrated any of the following health conditions: severe anemia (hemoglobin < 70 g/L), severe wasting (defined as weight-for-height z-score (WHZ) <−3 SD with respect to WHO 2006 standards [28]), bipedal edema, severe illness warranting hospital referral, congenital abnormalities that may interfere with growth, chronic medical conditions requiring frequent medical attention, known HIV infection of the index child or the child’s mother, ongoing use of micronutrient supplements, or current participation in another research study.

### 2.5. Randomization

Randomization was done by a statistician at the University of California Davis by assigning the study ID numbers to the four study arms, using a block randomization scheme with block lengths of four or eight. In the event that multiple siblings in the target age range resided in the same household, only the youngest was enrolled. In the case of twins, both twins were assigned to the same group and received all study-related interventions and follow-up, but only one was randomly selected for inclusion in the data analyses.

### 2.6. Study Interventions and Follow-Up

Children were assigned to one of four groups: (1) the preventive zinc (PZ) supplementation group, which received a daily preventive zinc dispersible tablet containing 7 mg zinc; (2) the MNP group, which received a daily preventive MNP containing 10 mg zinc, 6 mg iron and 13 other micronutrients; (3) the therapeutic zinc (TZ) supplementation group, which received 20 mg of zinc for 10 days for diarrhea treatment; or (4) the placebo control group, which received a daily placebo preventive powder. To ensure blinding across the 4 intervention groups, the PZ, MNP, and placebo groups received placebo therapeutic tablets for diarrhea and the TZ group received daily preventive placebo tablets [26]. In this sub-study, we focused only on the PZ, MNP, and placebo groups.

The zinc and placebo tablets were produced by Nutriset SAS (Malaunay, France). The MNP and placebo powder sachets were produced by DSM Fortitech Asia Pacific (Banting, Malaysia). Standard quality control procedures were used by the manufacturers to confirm the nutrient concentrations. The supplements were pre-labeled (by the manufacturer) with different numerical and alphabetical codes. Products were assigned specific colors (one color per intervention group) to ensure correct delivery to children in the respective study groups. In addition, each child, irrespective of the study group, was given low-osmolarity ORS sachets for diarrhea management as part of the diarrhea home treatment kit [26]. Supplements and ORS were provided at enrollment and replenished during weekly home visits, as needed. At enrollment, caregivers were instructed on how to administer the study products to their child, and field workers repeated these instructions every month during one of the weekly home visits. For the preventive dispersible zinc tablets, caregivers were instructed to dissolve one tablet with clean water or breastmilk and feed the resulting suspension to the child at least 30 min before or after a meal. Caregivers were instructed to mix the entire contents of an MNP or placebo powder sachet into a small amount of semi-solid food that the child could easily consume. Each child was visited weekly for 36 weeks unless lost to follow-up. During the weekly visits, caregivers were interviewed regarding the consumption of the intervention products. In addition, used and unused blister packages and sachets were collected to assess adherence.

### 2.7. Data Collection

All data were recorded electronically via customized data collection forms using CommCare-HQ (Dimagi, Boston, MA, USA) and deployed on portable Samsung tablets (Samsung Galaxy, Tab-4). At baseline, duplicate anthropometric assessments were recorded by trained anthropometric teams, using standardized procedures. Measurements included weight to the nearest 0.02 kg (SECA 383 balance) and recumbent length to the nearest 0.1 cm (SECA 416 length board). In the event that the duplicate measurements differed by >0.1 kg for weight, or by >0.5 cm for recumbent length and MUAC, a third independent measurement was taken. Means were computed using the two measures with the lowest absolute differences. Children who were severely wasted (WLZ < −3 SD) at baseline were excluded from participation and referred to the nearest health center or hospital. The anthropometric assessments were repeated after 18 weeks and at the endline (32–40 weeks). Anthropometric teams were systematically standardized [24,26]. During a total of four standardization sessions, the mean technical error of measurement (TEM) and coefficient of reliability for length were 0.38 cm and 97%, respectively.

For the purpose of evaluating biomarkers of nutritional status and health, ~7 mL samples of venous blood were collected by trained nurses from a convenience subsample of children (N = 760 for the parent study), using evacuated, trace element-free, 7.5 mL Lithium-Heparin tubes (Sarstedt AG & Co., Numbrecht, Germany). The heparinized samples were maintained at 4–8 °C in portable cooler boxes until they were transported to field laboratories for plasma processing within <8 h. In the field laboratory, the blood samples were centrifuged (PowerSpin Centrifuge Model LX C856; United Products & Instruments, Inc., Dayton, NJ, USA) at 1097× *g* (3100 RPM) for 10 min and the plasma was aliquoted into clear or amber microcentrifuge tubes (0.2–1.5 mL per tube), depending on the pre-planned analyses. Plasma samples were stored at −20 °C, and later shipped on dry ice to a permanent laboratory at the University of California, Davis, USA. The original goal was to analyze a total of 420 pairs of samples, from baseline and endline, from a total of 140 children per group, randomly selected from the list of children which plasma samples at both time points. However, some children did not have adequate plasma volumes (~200 µL) needed for the IGF1 and IGFBP3 assays. Hence, the final sample size for the sub-study (n = 112) for PZ, n = 141 for MNP and n = 155 for Placebo) was based on the number of children who had ~200 µL of plasma at both time points.

### 2.8. Patient and Public Involvement

Patients or the public were not involved in the design, conduct, reporting, or dissemination plans of our research.

### 2.9. Laboratory Analyses 

Determination of plasma IGF1 and IGFBP3 concentrations was performed at the University of Colorado, Boulder using an automated immune diagnostic system (IDS-iSYS Multi-Discipline Automated System; Immunodiagnosticsystems (IDS), East Boldon, UK). Blood samples collected at baseline and endline were analysed for IGF1 and IGFBP3 concentrations using IDS-iSYS IGF1 and IGFBP3 assays, with the IDS-iSYS Multi-Discipline Automated System (Immunodiagnostic Systems Holdings PLC, Boldon, UK). The LOD levels for IGF1 and IGFBP3 assays were 10 ng/mL and 80 ng/mL, respectively. The intra and inter assay CV% for the IGF1 assay were 3.4 and 6%, and for the IGFBP-3 assay were 2.5% and 5.4%, respectively. The IDS-iSYS was calibrated against a reference pediatric population and requires ~100 µL of plasma for both analytes. Plasma zinc was analyzed by inductively coupled plasma optical emission spectrophotometry (ICP-OES Agilent 5100 SVDV, Santa Clara, CA, USA) at the Children’s Hospital of Oakland Research Institute (CHORI, Oakland, CA, USA).

### 2.10. Data Entry and Analyses

A statistical analysis plan was developed and published prior to analyses [29] and was strictly followed to minimize bias. The groups’ identities were revealed only after the data analyses were completed and consensus was reached by the study investigators on the interpretation of the results. All analyses were performed with STATA statistical software, release 13 (StataCorp, Austin, TX, USA) and SAS version 9.4 (SAS Institute, Cary, NC, USA).

### 2.11. Definitions

The primary outcome variables were the concentrations of IGF1 and IGFBP3 at the endline (~36 weeks). The primary exposure variable was the intervention group as defined above. Standardized anthropometric measures based on the WHO Child Growth Standards were used in defining physical growth outcomes [28]. Stunting was defined as LAZ < −2 SD; and underweight as WAZ < −2 SD. Anemia was defined as hemoglobin < 110 g/L, and zinc deficiency as plasma zinc < 65 µg/dL after adjusting for inflammation as recommended by the BRINDA project [30].

### 2.12. Statistical Analyses

As mentioned above, the present analyses were restricted to children in PZ, MNP, and control groups. All analyses were performed on an intention-to-treat basis among children with available data [31]. In all analyses, the intervention group was considered the primary exposure variable. ANCOVA models were used to assess intervention differences in IGF1 and IGFBP3 concentrations at the endline, controlling for age at enrollment, region and sex. Models first assessed a global difference in treatment effect using a likelihood ratio test, and post-hoc pairwise differences were assessed subsequently in the event of a statistically significant global difference (global *p*-value < 0.05). To assess modifying effects of baseline IGF1 and IGFBP3 on the responses to physical growth, we constructed separate ANCOVA models with the midline (18 weeks) or endline (36 weeks) LAZ or WAZ as the outcome variables and intervention arm as the primary predictor variable. In addition, these models included interaction terms for the interactions between intervention and either baseline IGF1 or IGFBP3 as continuous variables. Significant interactions were defined as *p*-value < 0.1 for the interaction coefficient. Where necessary, marginal plots or two-way bar graphs with confidence intervals were plotted to visualize significant interactions. LOWESS plots or histograms were used to visualize associations and distributions of continuous variables. IGF1 and IGFBP3 data were log-transformed to normalize their distributions.

## 3. Results

### 3.1. Sampling and Baseline Data

**Figure 1:**
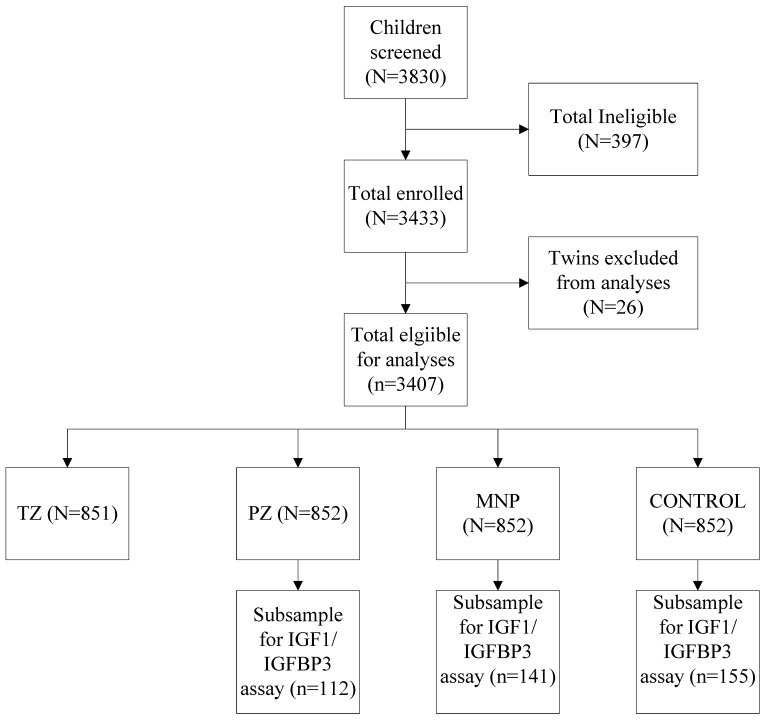
CONSORT Diagram indicating the selection of children into growth biomarker sub-study. Detailed CONSORT including an explanation of exclusions is published elsewhere [24,25].

Of the 3407 children enrolled in the parent study, we included children from the PZ (n = 112), MNP (n = 141), and control groups (n = 155) who had adequate plasma at both baseline and endline (Figure 1). At baseline, the mean age was 15.7 ± 5.0 months (Table 1), 40% of the children were stunted, and 46% were anemic. At baseline, median IGF1 and IGFBP3 concentrations were ~46 ng/mL and 2143 ng/mL.

### 3.2. Main Effects of PZ and MNP on IGF1 and IGFBP3

There were no main treatment effects on growth biomarker concentrations. At the endline, the geometric mean IGF1 concentration was ~39 ng/mL, with no differences across the groups (*p* = 0.99, Table 2). Similarly, there was no response in IGFBP3 to either the single-nutrient zinc supplement or the MNP supplement. The average endline mean IGFBP3 ranged from 2038–2075 ng/mL across the groups (*p* = 0.83). Consistent with the lack of response in IGF1 and IGFBP3, there was no main treatment effect on the molar IGF1:IGFBP3 ratio (*p* = 0.74).

### 3.3. Modifying Effect of Baseline IGF1 and IGFPB3 on LAZ and WAZ

**Figure 2:**
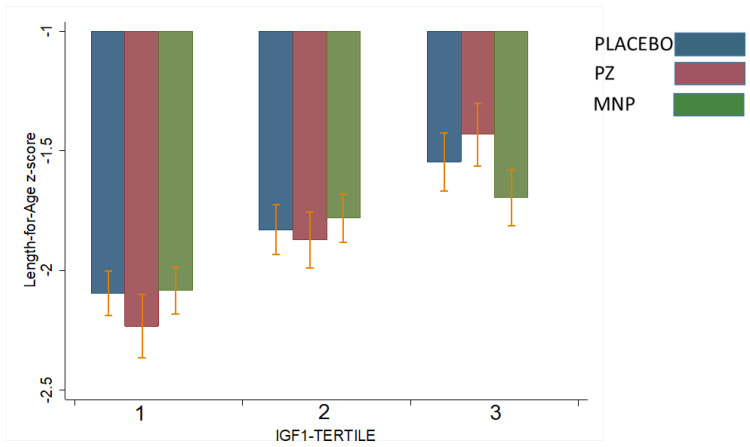
Effect-modification by baseline IGF1 of the response in length-for-age z-score (LAZ) at 18 weeks to zinc-alone or zinc-containing MNP in rural Laotian children. Bars with different letters are significantly different (*p*-value < 0.05).

**Figure 3:**
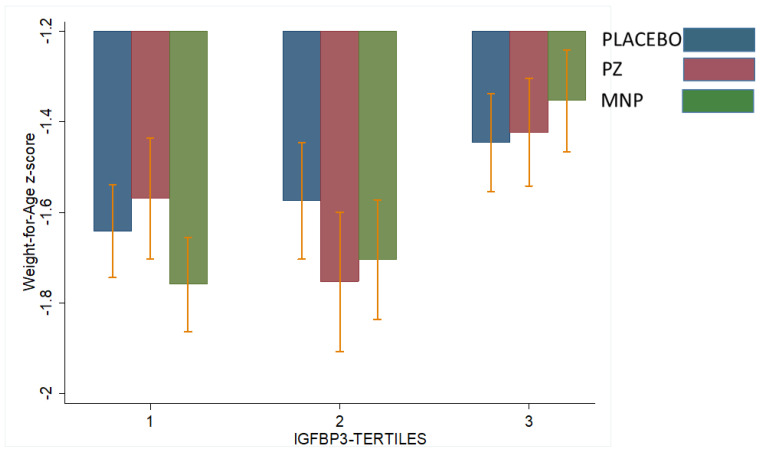
Effect-modification by baseline IGFBP3 of the response in weight for-age z-score (WAZ) after 36 weeks to zinc-alone or zinc-containing MNP in rural Laotian children. Bars with different letters are significantly different (*p*-value ≤ 0.05).

Table 3 shows the interactions between baseline IGF1 or IGFBP3 concentrations and modifying treatment response with respect to LAZ and WAZ at midline (18 weeks) or endline (36 weeks). Two significant interactions were observed. Baseline IGF1 modified the treatment response on LAZ at 18 weeks (*p* for interaction = 0.006, Table 3). Specifically, for children in the highest baseline IGF1 tertile, LAZ at 18 weeks in the PZ (−1.45) group was higher than the MNP (−1.70) and control (−1.55) groups (*p* = 0.01; Figure 2). This effect was not observed at 36 weeks. IGF1 did not modify the treatment effect on WAZ. However, baseline IGFBP3 modified treatment response on WAZ at 36 weeks (*p* of interaction = 0.06; Table 3). Specifically, for children in the lowest baseline IGFBP3 tertile, WAZ at 36 weeks in the PZ group (−1.55) was significantly higher than the MNP (−1.75) and control (−1.65) groups (*p* = 0.03, Figure 3). This effect was not observed at 18 weeks. IGFBP3 did not modify the treatment effect on LAZ. These interactive effects did not translate into statistically significant group differences in the molar IGF1:IGFBP3 ratio.

## 4. Discussion

In the present study, neither preventive zinc supplements nor MNP had an effect on IGF1 and IGFBP3 concentrations when provided for 9 months to 6–23 mo old children in rural Lao PDR. However, baseline levels of these biomarkers significantly mediated the linear and ponderal growth response to preventive zinc supplementation. Specifically, the highest response in linear growth to PZ was observed in children with the highest baseline IGF at 18 weeks; whereas the highest ponderal growth response to PZ was observed in children at 36 weeks among children with the lowest IGFBP3 at baseline.

Although there is a strong body of scientific evidence linking zinc supplementation to physical growth in children [3,4,5,6], the parent study did not find an impact on overall growth [24], nor did the present study find an effect on the growth factor biomarkers. Plausible reasons for this lack of effect of zinc supplementation on IGF1 and IGBPB3 may include (a) the dose of zinc used in this study and (b) a lack of sufficient growth potential in the study population. In the aforementioned Vietnamese study which found a positive response in IGF1 in children of similar age [23], a higher daily dose of 10 mg (instead of 7 mg) single-nutrient zinc supplement was used. Similarly, a study by Rocha et al. reported a positive response in both IGF1 and IGFBP3 after 3 months of supplementation with a daily zinc dose of 10 mg/d in a relatively older pediatric population (8–9 years) [32]. It is thus possible, that the zinc content of 7 mg in the single-nutrient zinc supplement was inadequate, and although the zinc dose in the MNP used in this study was 10 mg, it is generally accepted that the absorption and bioavailability of zinc from MNP is lower [33]. We found one study which reported a significant effect on IGF1 and IGFBP3 levels despite administering a zinc dose lower than used in the current study [34]. In this non-randomized pre-post study, by Alves et al., all children received a daily oral zinc dose of 5 mg, in addition to a baseline dose of 0.065 mg zinc per kilogram body weight given to all children. This study was however implemented in older children (6–8 years) and it is possible that the age differences may have played a role considering that IGF1 and IGFBP3 concentrations generally increase with age in young children [35,36].

Another possible reason for the lack of response in IGF1 and IGFBP3 could be that perhaps our study population had underlying conditions that inhibited IGF1 production in quantities necessary to stimulate growth. At baseline median IGF1 and IGFBP3 concentrations were ~46 ng/mL and 2143 ng/mL. These values are ~2–3 times lower than reference values reported in apparently healthy Turkish children 0–6 years of age [35] and in French children and adolescents 6–20 years [36]. Furthermore, the reference molar IGF1:GFBP3 ratio reported for children in the youngest age group in the French study (0.14) was approximately twice the value observed in this population [36], which might suggest that children in our study population may have had a relatively lower growth potential to begin with. However, in a Vietnamese population, in which a response in IGF1 (to zinc supplements) was observed, the reported IGF1 values at 1 and 5 months were about half the baseline concentrations observed in this population. Hence, it is unlikely that the lack of response is wholly attributable to low initial levels of these growth biomarkers. Additional studies on the diagnostic potential of IGF1 and IGFBP3 would improve understanding of the GH-IGF axis, and the mechanism underlying their effect on growth.

It has been documented that inherited hemoglobin disorders, which are highly prevalent in this population [37,38], interfere with the GH-IGF axis [39,40,41]. We previously reported that inherited hemoglobin disorders in this population adversely affected the response to MNP by increasing the risk of diarrhea infection [37]. However, this adverse effect was very small and it is unlikely that this may have played a role in the lack of response in IGF1, IGFBP3, and overall physical growth, although previous evidence suggests that diarrhea impairs linear growth [42]. Unfortunately, we did not have hemoglobinopathy data for all children in this subset, making it impossible to assess this potential interaction.

**Figure 4:**
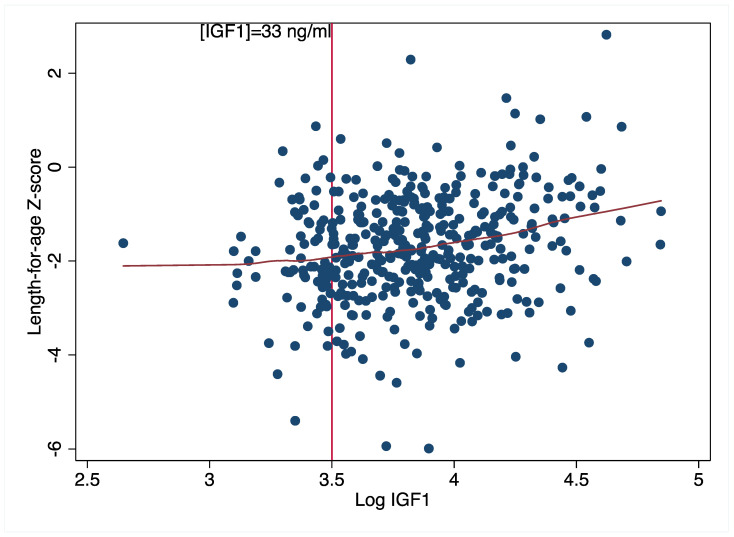
Association between baseline length-for-age z-scores and IGF1 concentrations.

Our findings provide some support for the role of the GH-IGF axis in the nutritional modulation of physical growth. Zinc supplementation improved linear growth among children in the highest IGF tertile and also improved ponderal growth among children in the lowest IGFBP3 tertile. Both high IGF1 and lower IGFBP3 suggest a higher IGF1 bioavailability. Hence, collectively, our findings suggest that zinc supplementation improved physical growth in the children with the highest physiologic growth potential at baseline. This is consistent with evidence from mouse models linking IGF1 bioavailability to linear growth [43]. As suggested by Dewey [44] the extent to which a nutritional intervention may have an impact may depend on whether individuals have the potential to benefit from an intervention or the potential to respond. Our findings with respect to IGFBP3 (but not IGF1) suggest that the most malnourished children were able to respond to the intervention, although this effect was no longer apparent at the endline. Our findings with respect to IGF1 are, however, somewhat contradictory. A plausible explanation is that there is possibly a minimum threshold of baseline IGF1 below which the nutritional modulation of linear growth is impaired. Our baseline data provided some support for this threshold effect (Figure 4). We observed that for IGF1 values below 33 ng/mL (Ln 3.5, Figure 4), LAZ values stayed consistent at ~−2 standard deviations below the reference mean; beyond this, we observed a steady rise in LAZ with increasing IGF1 values. Thus, it is possible that the children in this population did not produce enough IGF1 to stimulate physical growth. Unfortunately, the lack of reference values for IGF1 and IGFBP3 makes it challenging to define potential deficiencies in these growth biomarkers. However, because there were few children with IGF1 values below this threshold (n = 66), this distribution must be interpreted with caution.

**Figure 5:**
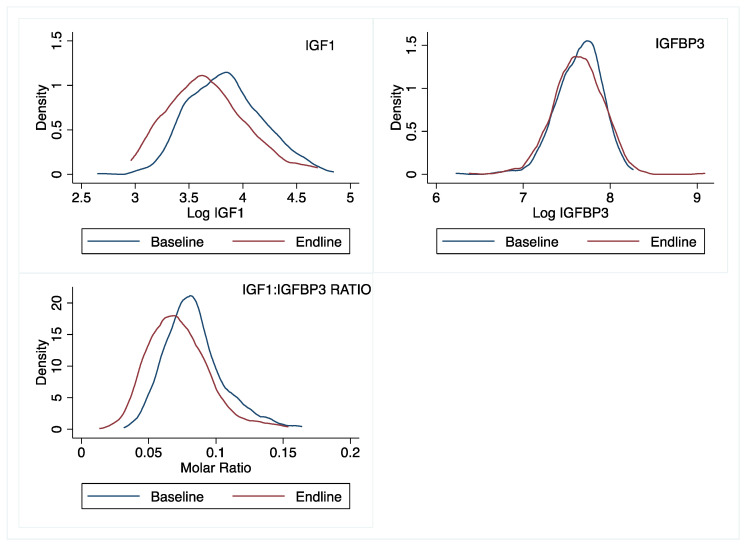
Distributions of IGF1, IGFBP3, and molar IGF1:IGFBP3 ratio at baseline and 36 weeks later in rural Laotian children.

Finally, it is interesting that the modifying effects of IGF1 and IGFBP3 were apparent at different time points; the mediating effect of IGF1 (on linear growth) and IGFBP3 (on ponderal growth) were apparent at ~18 weeks and 36 weeks, respectively. This pattern may be related to observed longitudinal shifts in the distribution of these markers (Figure 5). In this population, we observed that by endline (~36 weeks), the IGF1 distribution had shifted to the left, relative to baseline concentrations (Figure 5). Over the same period, the baseline and endline IGFBP3 distributions were nearly identical. Although we did not collect IGF1 and IGFBP3 data at 18 weeks, these shifts would suggest that IGF1 bioavailability declined over time, and perhaps explains why IGF1 modified linear growth at 18 weeks but not later (at 36 weeks). The lack of a sustained linear growth responses through 36 weeks is likely a combination of both the declining IGF1 values and the inhibitory effects of excess, unbound IGFBP3 [45]. In mouse models, excess IGFBP3 has been associated with reduced pre- and postal natal growth [46,47,48]. This inhibitory effect by IGFBP3 may partly explain why only children in the first (but not higher) IGFBP3 tertile demonstrated a growth response to a single-nutrient zinc supplement. It is also worth mentioning that the children in our study group are around the period where there is a transition in growth regulation, from insulin-driven IGFs, to end endogenous pulsatile GH [49]. Thus, it is possible that variations in this transition may have contributed to the outcomes observed in this study.

A weakness of this study is the lack of growth biomarker data at 18 weeks. In retrospect, it would have been more appropriate to collect this data to allow for a more accurate determination of the changes in IGF1 and IGFBP3 over time, and their association with growth and age. A strength of this study is the individualized randomization protocol and the sample size. To our knowledge, this is the largest randomized trial to date assessing the impact of different zinc formulations on these growth biomarkers, in a population with a high prevalence of stunting.

In conclusion, our study suggests that although IGF1 and IGFBP3 did not directly respond to zinc and MNP, both biomarkers may play a critical role in mediating the physical growth response to zinc supplementation.

## Figures and Tables

**Table 1:** Demographic data, nutritional status, and growth biomarker concentrations at baseline in intervention groups.

Variable	PZ (n = 112)	MNP (n = 141)	Placebo (n = 155)
Mean Age (months)	15.7 ± 5.3	15.7 ± 4.9	15.7 ± 5.3
Males (%)	55 (48.3)	76 (52.4)	85 (54.8%)
LAZ	1.78 ± 1.03	1.77 ± 1.06	1.68 ± 1.09
WAZ	1.48 ± 1.01	1.46 ± 1.02	1.38 ± 1.00
Stunting (%)	51 (45.1)	58 (40.0)	50 (32.3)
Anemia (%)	52 (45.6)	70 (48.3)	73 (47.1)
Zinc Deficiency * (%)	87 (78.4)	88 (73.3)	95 (81.2)
Growth Biomarker			
IGF1	47.6 (47.1, 53.8)	46.8 (44.2, 49.5)	46.1 (43.4, 48.7)
IGFBP3	2097 (1996–2257)	2094 (2009, 2183)	2131 (2046, 2215)
Molar IGF1:IGFBP3 ratio	0.08 (0.07, 0.09)	0.08 (0.07, 0.09)	0.08 (0.07, 0.09)

* Results are shown as mean ± SD or N (%); For growth biomarkers, results are shown as geometric mean (95% confidence interval). Plasma zinc concentration < 65 mg/dL and adjusted for inflammation as recommended by the BRINDA project [30].

**Table 2:** Effects of 32—40 weeks of supplementation with daily preventive zinc supplements, daily MNP on Insulin-like Growth Factor 1 (IGF1) and Insulin-like Growth Factor 1 Binding Protein (IGFBP3) among rural Laotian children.

	PZ (n = 112)	MNP (n = 141)	Placebo (n = 155)	*p*-Value *
IGF1 at endline, ng/mL	39.1 (37.0, 41.2)	39.2 (37.4, 41.0)	39.0 (37.2, 40.8)	0.99
Change in IGF1, ng/mL	−7.7 (−10.3, −5.1)	−7.8 (−10.1, −5.6)	−8.2 (−10.3, −6.0)	0.96
IGFBP3 at endline, ng/mL	2037.6 (1946.4, 2128.8)	2075.5 (1995.5, 2155.5)	2055.0 (1997.8, 2132.5)	0.83
Change in IGFBP3, ng/mL	−76.2 (−162.1, 9.6)	−3.6 (−77.8, 70.6)	−16.2 (−88.3, 55.9)	0.42
Molar IGF1:IGFBP3 at endline	0.073 (0.069, 0.077)	0.071 (0.068, 0.074)	0.072 (0.068, 0.075)	0.74

* Values adjusted for age, sex, district baseline values of IGF1, IGFBP3, length-for-age z-score, weight-for age z-score, and weight –for-length z-score.

**Table 3:** Interaction between baseline IGF1 or IGFBP3 and zinc supplementation in predicting physical growth at 18 weeks and 36 weeks.

Time Point/Baseline Factor	LAZ	WAZ
18 weeks		
IGF1	0.006	0.329
IGFBP3	0.110	0.535
36 weeks		
IGF1	0.32	0.96
IGFBP3	0.38	0.06

## Data Availability

All data is available at: https://osf.io/5bq9c/.
